# Correlated analytical and functional evaluation of higher order structure perturbations from oxidation of NISTmAb

**DOI:** 10.1080/19420862.2022.2160227

**Published:** 2023-01-22

**Authors:** Tsega L. Solomon, Frank Delaglio, John P. Giddens, John P. Marino, Yihua Bruce Yu, Marc B. Taraban, Robert G. Brinson

**Affiliations:** a Institute for Bioscience and Biotechnology Research, National Institute of Standards and Technology and the University of Maryland, Rockville, Maryland, United States; b Bio- and Nano-Technology Center, University of Maryland School of Pharmacy, and Institute for Bioscience and Biotechnology Research, Rockville, Maryland, United States

**Keywords:** low-field water NMR relaxometry, 2D NMR, biologics, NISTmAb, higher order structure, surface plasmon resonance, antigen binding

## Abstract

The clinical efficacy and safety of protein-based drugs such as monoclonal antibodies (mAbs) rely on the integrity of the protein higher order structure (HOS) during product development, manufacturing, storage, and patient administration. As mAb-based drugs are becoming more prevalent in the treatment of many illnesses, the need to establish metrics for quality attributes of mAb therapeutics through high-resolution techniques is also becoming evident. To this end, here we used a forced degradation method, time-dependent oxidation by hydrogen peroxide, on the model biotherapeutic NISTmAb and evaluated the effects on HOS with orthogonal analytical methods and a functional assay. To monitor the oxidation process, the experimental workflow involved incubation of NISTmAb with hydrogen peroxide in a benchtop nuclear magnetic resonance spectrometer (NMR) that followed the reaction kinetics, in real-time through the water proton transverse relaxation rate *R*_2_(^1^H_2_O). Aliquots taken at defined time points were further analyzed by high-field 2D ^1^H-^13^C methyl correlation fingerprint spectra in parallel with other analytical techniques, including thermal unfolding, size-exclusion chromatography, and surface plasmon resonance, to assess changes in stability, heterogeneity, and binding affinities. The complementary measurement outputs from the different techniques demonstrate the utility of combining NMR with other analytical tools to monitor oxidation kinetics and extract the resulting structural changes in mAbs that are functionally relevant, allowing rigorous assessment of HOS attributes relevant to the efficacy and safety of mAb-based drug products.

## Introduction

Monoclonal antibodies (mAbs) have become the leading class of protein therapeutics for a wide range of illnesses. While their versatility and success in targeting diseases have made mAbs the leading biologic platform in the pharmaceutical industry, characterization of these large and complex proteins remains a challenge. Quality assessment of mAbs requires establishing metrics for critical quality attributes (CQAs) that are considered essential for biological activity and safety during the drug manufacturing processes, storage, and patient administration.^1,2^ Higher order structure (HOS), from secondary to quaternary structure, is a key CQA that determines the efficacy and safety of mAb drugs.^3^ Perturbations to mAb HOS, which can result from many causes such as misfolding or chemical modifications, can hinder proper function of the drug and potentially elicit an unintended immune response, thereby compromising the efficacy and safety of the therapeutic.

During development, knowledge of potential product degradation pathways is also necessary to determine drug shelf life and resistance to various stresses. However, real-time stability studies often provide insufficient information about the degradation products that may be observed during the life cycle of a product.^4,5^ To facilitate acquisition of this essential product knowledge, forced degradation studies using approaches such as oxidation, temperature, pH, light, freeze-thaw, and agitation can provide important molecular information and indicators of possible issues that may arise during, for example, transportation, drug preparation, or administration. These studies have therefore become a critical component of the drug development process.^6,7^

One commonly applied forced degradation method is chemical oxidation. Oxidation of amino acids is one of the most prevalent post-translational chemical modifications that can occur in the cellular environment, during formulation of the therapeutic protein or in storage.^8^ A number of residues, including methionine (Met), cysteine, tryptophan, and histidine, are susceptible to oxidation in the presence of reactive oxygen species (ROS).^9^ Met oxidation, in particular, has been linked to alteration in mAb activity and stability, which has implications for drug quality and efficacy. This feature has made the mAb oxidation stress study a common pharmaceutical tool to evaluate the developability of drug candidates.^10^ One common oxidation method is to treat a mAb with hydrogen peroxide, a common ROS that primarily oxidizes Met residues via a nucleophilic substitution of the side-chain thiol to produce methionine sulfoxide (MetO) and in extreme excess cases, Met sulfone.^11^ Common analytical methods used to evaluate chemical oxidation include liquid chromatography (LC), cation exchange chromatography, and mass spectrometry.^10,12–14^ While these methods provide powerful tools for identifying and quantifying oxidation sites, in general they do not provide a readout of the effect of oxidation on the HOS of the drug. The one exception is hydrogen-deuterium exchange mass spectrometry, which can be correlated to HOS.^15^

Binding assays such as surface plasmon resonance (SPR) can provide an indirect readout of HOS perturbations, as a measure of decreased binding affinity to an antigen or receptor.^16^ As the level of MetO increases, the resulting SPR data can provide a readout in terms of reduced binding affinity.^16,17^ Recently, a rapid SPR assay was developed to quickly detect Met oxidation in both the antigen-binding fragment (Fab) and Fc domains.^18^ Using a model therapeutic IgG1κ mAb, NISTmAb,^19,20^ specific molecular probes were used to assess the binding to one region in the Fc (Protein A) and two regions in the Fab (Protein L, F peptide). The chemical oxidation was performed freely in solution or while NISTmAb was bound to Protein A. Since Protein A and the F peptide binding sites are proximal to Met residues in mAbs, SPR allowed for detection of low levels of oxidation that interfered with binding. By contrast, Protein L binds to the variable region of Fab light chain (V_L_), which is distal to any Met residue and therefore was not a robust reporter of the oxidation status of the mAb.

One limitation of analytical and functional assays is the lack of traceability to a high-resolution HOS method. Among the high-resolution biophysical methods, nuclear magnetic resonance spectroscopy (NMR) has become a well-established technique that provides atomic-level structural fingerprints, which can be used to evaluate structural changes in biological therapeutics. Previous applications of high-field NMR have shown the utility of gradient-selected 2D ^1^H-^13^C methyl correlation spectrum for providing a precise fingerprint of intact mAb reference molecule, NISTmAb, at natural abundance.^21,22^ This method has been successfully applied to HOS assessment of forced degradation studies using chemical oxidation, including studies on interferon alpha-2a,^23,24^ an IgG1 Fc domain,^25^ an IgG1,^26^ and the intact NISTmAb.^27^ Additionally, 2D ^1^H-^13^C NMR of the aliphatic region has been successfully applied to the quantification of MetO for small peptides and mAbs.^28^ In general, 2D NMR assessment affords specific HOS information and has been shown to be sensitive to low levels of oxidation.

Another emerging analytical technology that to date has only been sparingly applied in a pharmaceutical context is water proton NMR (*w*NMR) relaxometry implemented using time-domain, low-field benchtop instruments. While this is a low-resolution technique, recent work has demonstrated its general utility for use in pharmaceutical CQA assessment such as measurement of aggregation of biological therapeutics including mAbs.^29,30^ The *w*NMR method involves measuring the effect of HOS change of a solute on the proton transverse relaxation rate of water used as the solvent (*R*_2_(^1^H_2_O)). This rapid *R*_2_(^1^H_2_O) measurement involves no sample handling or consumption, and data can be collected noninvasively while the sample is within pharmaceutical vials.^31^ Compared to other current analytical methods such as size-exclusion chromatography (SEC), microflow imaging, and dynamic light scattering, *w*NMR afforded greater sensitivity to distinguish different levels of aggregation in mAb products, demonstrating the potential advantages of this simple, rapid measurement technique for monitoring additional mAb modifications that affect HOS-relevant CQAs.^30,32^

Here, we present a mAb oxidation case study that demonstrates the practical application of synergistically linking high-field NMR with lower resolution analytical and biophysical methods and functional assays to enable a correlated HOS assessment. Specifically, the time-dependent oxidation of Met by hydrogen peroxide in the model therapeutic, NISTmAb, was monitored by *w*NMR in conjunction with orthogonal analytical methods, including high-field NMR, thermal unfolding (Tycho), and SEC. These analytical results were then correlated with binding data from a rapid SPR assay.^18^ The observation of HOS variance that correlates with stability and functional changes underscore the utility of combining 2D ^1^H-^13^C methyl NMR with other analytical tools to yield a more precise evaluation of mAb candidates during pharmaceutical development.

## Results

### Real-time monitoring of mAb oxidation via wNMR

**Figure 1.**
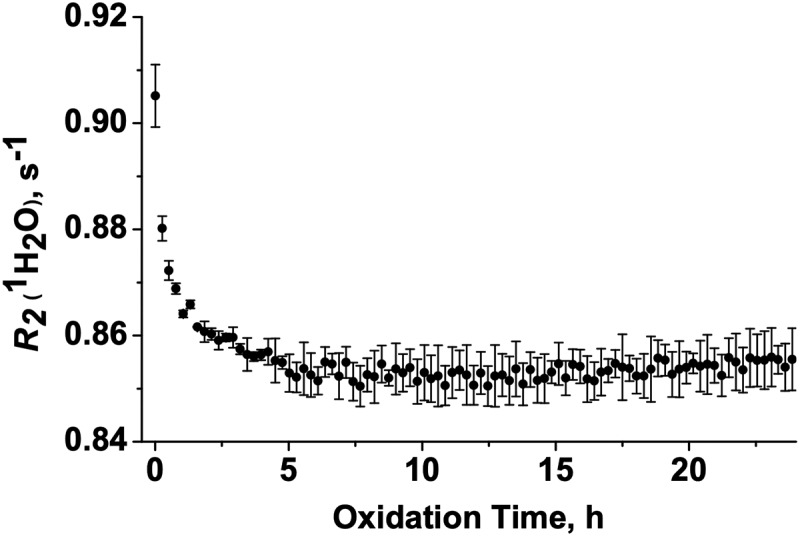
Monitoring NISTmAb oxidation kinetics using low-field benchtop NMR by means of the *w*NMR technique. Plot of water proton transverse relaxation rate, *R*_2_(^1^H_2_O), as a function of time after addition of H_2_O_2_ to the sample. Error bars represent the standard error for the average of two oxidation experiments.

Met oxidation of NISTmAb was monitored in real-time by means of the *w*NMR technique in a low-field benchtop NMR instrument using measured water proton transverse relaxation rates (*R*_2_(^1^H_2_O)) over time. Since the labile protons of protein backbone amides and of the side chains of polar amino acids exchange with water in aqueous solvent, the relaxation property of water protons can be used as a surrogate reporter of protein dynamic behavior and/or structural organization. The sensitivity of *R*_2_(^1^H_2_O) to mAb aggregation has been previously demonstrated, suggesting that the parameter may also respond to other mAb modifications.^30^ During the 24 `h time course of chemical oxidation of NISTmAb with hydrogen peroxide, *R*_2_(^1^H_2_O) was measured every 15 min, and aliquots were removed at varying time points in the course of the reaction to allow for more in-depth analysis by other analytical methods (Figure 1). A repetition of this experiment was then performed, during which aliquots were not removed to allow for undisturbed *R*_2_(^1^H_2_O) measurement (**Figure S5B)**. The average *R*_2_(^1^H_2_O) plot of two oxidation experiments (Figure 1) showed a nonlinear reduction in *R*_2_(^1^H_2_O) where the value of *R*_2_(^1^H_2_O) decreases from 0.91 to 0.85 s^−1^ in the first 5 h of oxidation and remained stable for the rest of the oxidation time. We note here that intensive method optimization was necessary to minimize experimental influence and error while measuring *R*_2_(^1^H_2_O) during the course of NISTmAb oxidation. Details of the optimization process are reported in the Supplementary Information (Supplementary Methods, **Figures S1–S6**).

### Detection of Met oxidation by 2D NMR fingerprinting

**Figure 2.**
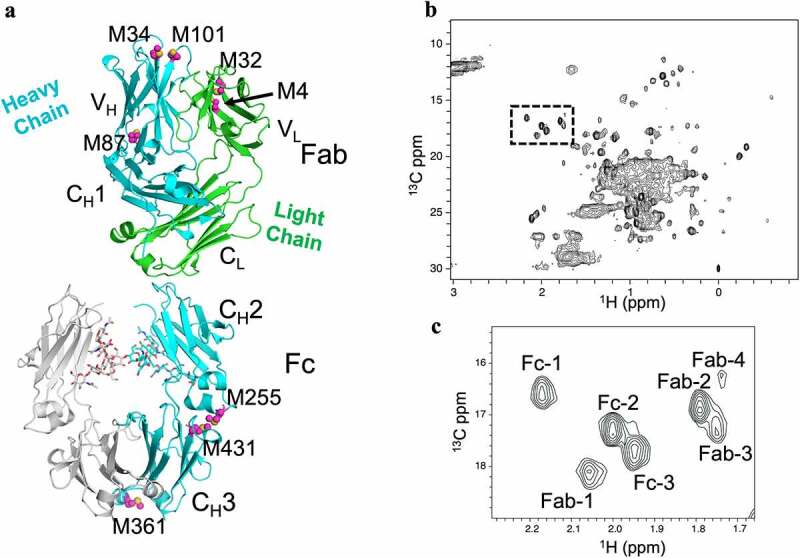
Methionine residues in NISTmAb. (a) Crystal structures of the Fab (PDB 5K8A) and Fc (PDB 5VGP) regions of NISTmAb showing positions of Met residues (magenta) in the Fab heavy chain (cyan), light chain (green) and Fc region (cyan). (b) The 2D ^1^H-^13^C methyl gHSQC spectra, collected at 600 MHz and 50°C, with Met methyl signals boxed. (c) Expanded view of the Met region boxed from panel B distinguishing signals arising from Met residues in the Fab and Fc fragments.

To analyze the impact of the extent of Met oxidation on HOS, 2D ^1^H-^13^C methyl gradient-selected, sensitivity-enhanced heteronuclear single quantum coherence (gHSQC) spectra were acquired at 50°C and 600 MHz for sample aliquots taken at ten time points along the oxidation time course and compared to an unoxidized reference NISTmAb sample. NISTmAb contains eight Met residues, three in the Fc region and five in the Fab region with three Met residues in the Fab heavy chain and two in the light chain (Figure 2a). The 2D methyl gHSQC spectrum of NISTmAb shows the methyl chemical shifts of seven Mets, between ^1^H 2.2–1.7 ppm and ^13^C 16–18.5 ppm, dispersed from the rest of the methyl signals (Figure 2b, boxed). Spectral alignment of the intact NISTmAb with those of the Fc and Fab enabled designation of these seven resonances: three peaks correspond to the Fc region and four to the Fab region (**Figure S7**). Further specific Met assignments are not available.

**Figure 3.**
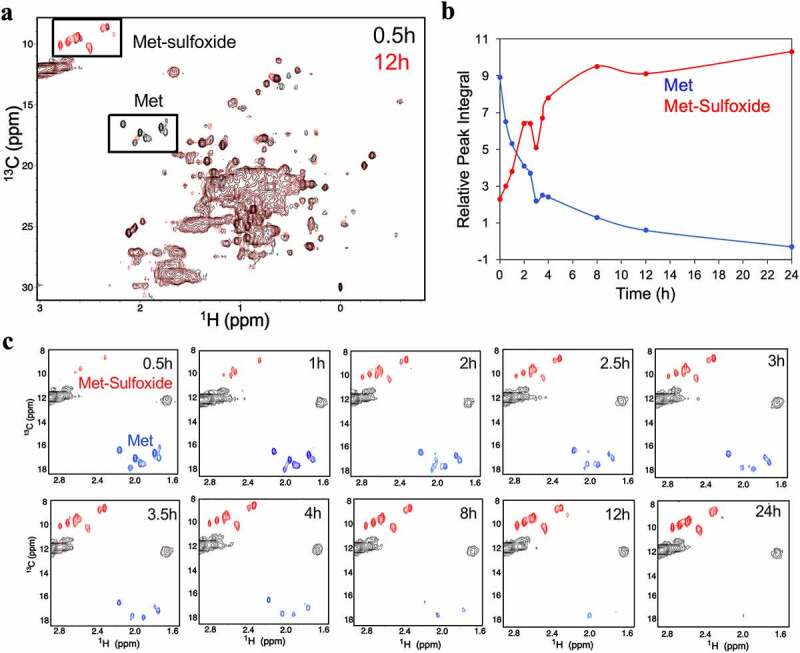
The time-dependent oxidation of methionine residues in NISTmAb detected via 2D ^1^H-^13^C methyl gHSQC spectra, collected at 600 MHz and 50°C. (a) Overlay of the 2D ^1^H-^13^C methyl gHSQC spectra of 0.5 h (black) and 12 h (red) oxidation time point aliquots. (b) The relative peak integral of Met and MetO regions as a function of oxidation time. (c) The Met and MetO regions of the ^1^H-^13^C Methyl HSQC spectra of each time point aliquots of NISTmAb oxidation.

**Figure 4.**
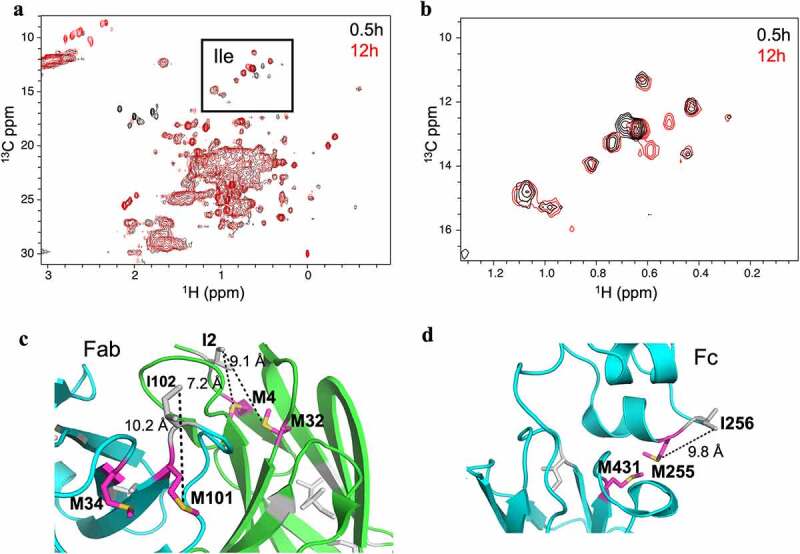
Methionine oxidation perturbs local structure. (a) Overlay of the 2D ^1^H-^13^C Methyl gHSQC spectra of 0.5 h (black) and 12 h (red) oxidation time point aliquots with Ile region boxed. (b) Expanded view of the Ile region boxed in panel (a). (c) Expansion of the NIST-Fab crystal structure showing Ile residues (gray) that are in proximity of methionine residues (magenta). (d) Expansion of the NIST-Fc crystal structure showing Ile residues (gray) that are in proximity of methionine residues (magenta). For panels c and d, the distance between Ile methyl group and proximal methionine thiol is provided and represented as dashed lines.

The ^1^H, ^13^C methyl gHSQC spectra of hydrogen peroxide oxidized samples demonstrated a gradual decrease of Met signal intensities during the course of oxidation (Figure 3). The Met signals declined significantly within the first 4 h of oxidation, but a few broad signals continued to be detected until 12 h of oxidation. As oxidation progressed, new signals corresponding to MetO appeared in a distinct region between 2.4–2.8 and 8–10 ppm in ^1^H and ^13^C dimensions, respectively. Although initially broad and fewer than expected, MetO signals are detected within 0.5 h of oxidation. With increasing oxidation time, the intensity of MetO signals increased, and eight correlations were detected after 3 h of oxidation. As would be expected, the plot of peak integrals of the Met and MetO signals shows the decline of Met signals is inversely proportional to the buildup of MetO signals (Figure 3b). Based on this peak integral evaluation, it is estimated that 70% of the Met oxidation takes place within the first 4 h of incubation with hydrogen peroxide. In addition to the Met and MetO signals, visual inspection by spectral overlay shows chemical shift changes in other regions of the spectra, most notably in the dispersed isoleucine (Ile) peaks, near ^1^H 0.5 ppm and ^13^C 12 ppm (Figure 4a, b). Consistent with the 2D NMR data, the Fab and Fc crystal structures show a number of Ile residues that are proximal to Met residues (Figure 4c, d). The measured distance between the Ile methyl groups and oxidizing Met thiol groups ranged between 7.2 and 10.2 Å, which is close enough to the site of oxidation to potentially induce the chemical shift perturbations detected in the methyl spectra. This observation suggests that formation of MetO affects the local structure surrounding the oxidized Met, consistent with a previous report that looked at oxidation of an Fc domain.^25^

### Classification of NISTmAb oxidation series with principal component analysis

**Figure 5.**
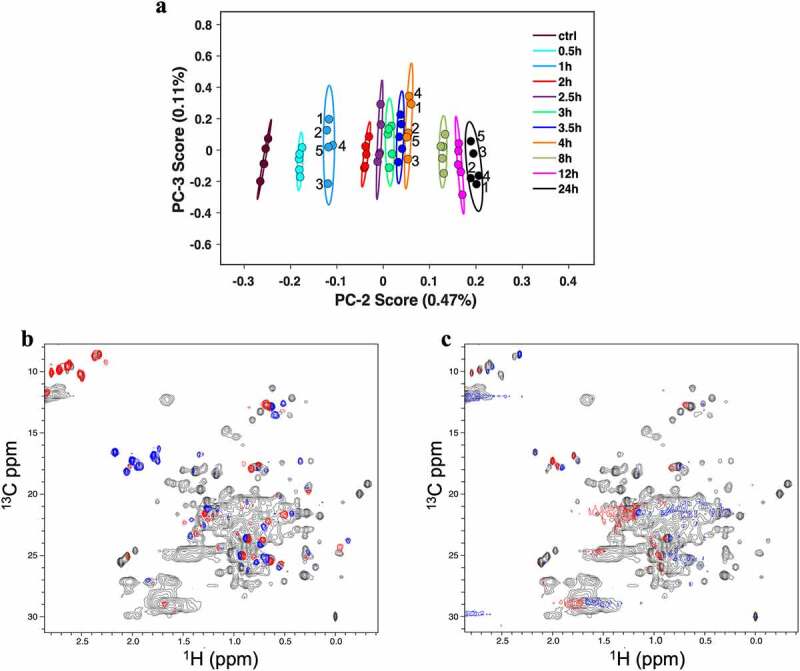
PCA clusters 2D ^1^H-^13^C methyl gHSQC spectra based on oxidation time course. (a) PCA score plot of PC-2 and PC-3 from analysis of the entire methyl region of each time point aliquots. Replicate experiments have the same color. The representative labeled points indicate experimental replicates, to show random distribution in PC-3. (b) The spectral loading plot of the second component, PC-2, (red and blue contours) overlaid on the average series spectrum PC-1 (gray). (c) The spectral loading plot of the third component, PC-3, (red and blue) overlaid on the average series spectrum PC-1 (gray). The red and blue contours reflect positive and negative intensity, respectively. The average series spectrum (gray) in Panel a and b, PC-1, is the spectral loading plot of the methyl region from the 2D ^1^H-^13^C methyl gHSQC spectra of all the oxidation time point aliquots, which consist of 5 replicate experiments for each time point aliquot.

A comprehensive analysis of oxidation-induced spectral change can be achieved by using principal component analysis (PCA) on the 2D ^1^H-^13^C methyl HSQC spectra series. The PCA approach is well established for analyzing variance in large NMR datasets and reduces the multivariate nature of the NMR measurement into a set of decoupled, orthogonal variables that are known as principal components (PC).^22,33^ As it has become established practice to perform PCA on raw 2D datasets without normalization by column centering, the first PC corresponds to the average spectra. As such, PC-1 can be considered as the average spectrum for the entire spectral series, so peaks corresponding to both Met and MetO signals are observed in the average PC-1 spectral loading plot of the oxidation spectral series. The PC-2 and PC-3 score plot clusters replicate spectra and distinguish the oxidation series in the order of oxidation time (Figure 5a). In particular, the spectra from samples taken at different oxidation time points show significant separation along PC-2 scores, whereas replicate spectra vary along PC-3 scores. The greatest change in PC-2 score is detected within the first 4 h of oxidation, consistent with the observed *R*_2_(^1^H_2_O) difference from the *w*NMR measurements. The dispersion of replicate spectra along PC-3, however, do not have a specific order within a class or pattern across the different oxidation time samples, suggesting PC-3 is mostly capturing experimental variation among the five replicates.

When considering the spectral loading plots, the PC-2 loading plot clearly extract spectral changes in NISTmAb that relate to the extent of oxidation (Figure 5b). PC-3 and higher components only reflect experimental variation and noise contributions (Figure 5c). The observation of a select number of chemical shift changes in the PC-2 plot and the spectral alignment of 0.5 and 12 h oxidized samples indicate that the overall fold of the mAb fragments remain intact, and structural changes resulting from Met oxidation can be considered localized. In addition to highlighting the Met and MetO signal changes, the loading plot revealed chemical shift perturbations in other regions of the spectra, suggesting that the structural effects extend beyond the chemical change to the Met residues.

Even without resonance assignment, alignment of PC-2 loading plot with the ^1^H-^13^C methyl gHSQC spectra of NIST-Fab and NIST-Fc fragments enable designation of the source of spectral variation to each region of the NISTmAb. For example, when inspecting the Ile region of the aligned spectra, two Ile residues from Fab region and one Ile residue from Fc region are affected by Met oxidation (**Figure S8**). The two Fab Ile signals align with negative contours from the PC-2 loading plot, signifying that the change in methyl peak position likely arose due to the new positive signals that are observed in the region. The Fc Ile signal, however, aligns to a mixed-phase signal of positive and negative contours indicative of change in line-width and a small drift in peak position. This manual inspection of the PC-2 loading plot indicated the Fab Ile methyl signals shift more significantly than the Fc Ile.

To investigate whether the observed effect in PC space was due primarily to contributions from the Met and MetO regions, PCA was performed with these two regions excluded to determine if spectral clustering depended on the Met and MetO resonances (**Figure S9**). The resulting score plot of PC-2 versus PC-3 showed spectra clustering and separation of oxidation series that closely matched that of the full methyl spectra analysis (Figures 4A**, S9B**). Correlation analysis of the PC-2 scores of the entire methyl region and truncated methyl region excluding Met and MetO signals reflected a fairly linear relationship with a correlation constant, ρ, of 0.978 (**Figure S9C**). This observation suggests that Met oxidation induces structural changes that are readout by neighboring methyl groups.

### Assessment of thermal stability and higher molecular weight species

**Figure 6.**
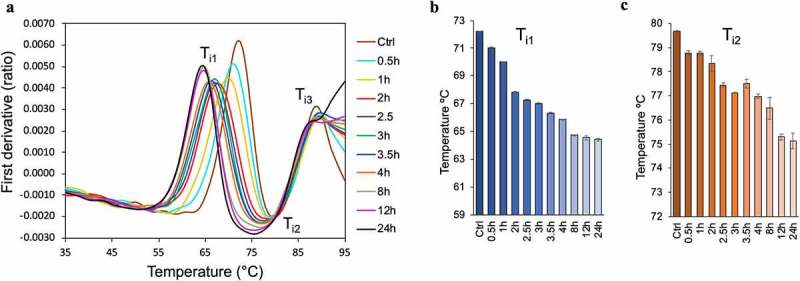
Measurement of unfolding transitions using intrinsic tryptophan fluorescence. (a) First derivative ratio of fluorescence at 350  and 330 nm showing inflection points correspond to unfolding events of: C_H_2 domain of Fc (T_i1_), C_H_3 domain of Fc (T_i2_), and Fab domain (T_i3_). (b) Bar graph representation of temperature change for the first inflection point, T_i1_. (c) Bar graph representation of temperature change for the second inflection point, T_i2_. Error bars represent the standard error from the average of two experiments.

The thermal stability of oxidized samples was assessed using a NanoTemper Tycho instrument, which estimates relative thermal unfolding transitions by measuring the intrinsic fluorescence of tryptophan at 350  and 330 nm over a span of 3 min from 35°C to 95°C. The first derivative plot of the fluorescence ratio (330/350 nm) detects three inflection temperatures, T_i1_, T_i2_, and T_i3_, for a NISTmAb reference, corresponding to the unfolding of the C_H_2 domain of Fc, C_H_3 domain of Fc, and Fab region, respectively.^34^ The thermal unfolding transitions of the NISTmAb oxidation series shift for T_i1_ and T_i2_, where the inflection temperatures decrease with increasing oxidation time. T_i1_ declines from 72°C to 65°C in the first 8 h of oxidation, and T_i2_ decreases from 80°C to 75°C in the first 12 h (Figure 6). The notable discontinuity in the decrease of T_i2_ between 3 and 4 h of oxidation perhaps reflects conformational changes in C_H_3 domain of Fc that do not necessarily increase solvent exposure of buried tryptophan. While an obvious temperature drift is not detected for T_i3_, the first derivative plot shows significant change in the transition of T_i3_ where the inflection is steadily lost with increasing oxidation time, and the profile appears to move upward instead of toward a minimum. The loss of smooth transition in T_i3_ is indicative of NISTmAb instability and aggregation, reflecting the decline in sample quality with increasing Met oxidation.

Based on the observation of aggregation at higher temperatures in the Tycho measurements, SEC was used to determine whether oxidation induced a shift in soluble aggregates at 25°C. SEC analyses showed that the level of monomeric, lower molecular weight fragments, and higher molecular weight species, such as soluble oligomers, do not vary significantly during the course of oxidation at the experimental condition (**Table S1**). The size variance observed in monomeric and higher molecular weight species of the oxidation series are within two standard deviation units of previously reported intrinsic heterogeneity of NISTmAb reference (PS-8670), which is below the three standard deviation threshold of significance.^35^ Thus, the observed aggregation by Tycho inflection profile T_i3_ indicates that this was likely caused by elevated temperatures.

### Assessment of mAb binding function

**Figure 7.**
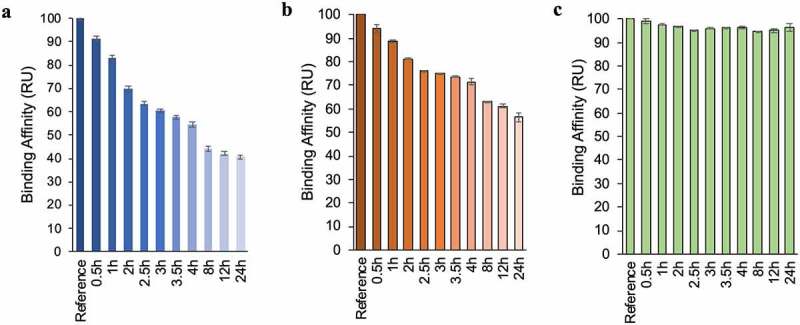
Ligand binding assessment of oxidized NISTmAb via SPR over the oxidation time course. (a) Relative binding affinity of protein A. (b) Relative binding affinity of F peptide. (c) Relative binding affinity of Protein L. Error bars represent the standard error from the average of two experiments.

Met oxidation in mAbs can alter biological activity by diminishing ligand binding to both the Fc and Fab regions.^18,36^ Here, to correlate the NMR and stability methods to a functional change, the binding capacity of oxidized NISTmAb was assessed by three binding partners that are known to have distinct binding sites on mAbs. Two of the partners are bacterial proteins, Protein A and Protein L, which bind to the Fc domain and to the variable region of the Fab light chain, respectively.^37–39^ The third is a synthetic peptide, F peptide, which binds to the complementary-determining region (CDR) of the Fab heavy chain. Oxidized NISTmAb binding to Protein A, Protein L, and F peptide was measured with an SPR assay where ligands are immobilized to a sensor chip. The SPR results showed that binding to Protein A and F peptide were sensitive to Met oxidation, causing a decline in affinity with increasing oxidation time. However, the binding affinity of protein L remained mostly unaffected (Figure 7). Relative binding assessment demonstrated that within 4 h of NISTmAb oxidation, binding affinity to Protein A decreased by 50% and binding to F peptide by 30%. Although binding capacity to both ligands continued to decrease until 24 h of oxidation, the first 4 h exhibit the fastest loss in binding affinity, which directly correlates to the results of *R*_2_(^1^H_2_O), 2D NMR, and Tycho measurements.

Inspection of the crystal structure of the Fab region, PDB 5K8A, and Fc region, PDB 5VGB, provide insight on the observed binding affinity change as it relates to the vicinity of Met residues relative to the ligand binding sites (**Figure S10**).^40,41^ Two of the Met residues in the Fab chain, M4 and M101, are surface exposed and part of the CDR through which the F peptide binds. Residues M255 and M431 in the Fc domain are also surface-exposed sites on the interface of C_H_2 and C_H_3 where protein A binds. However, residues M4 and M32 in the variable light chain are located farther from the Protein L binding site, which is consistent with the observation of unperturbed binding of oxidized NISTmAb to protein L. This variance of Met oxidation impact on binding affinity is consistent with the localized spectral perturbations from Met modification in the 2D ^1^H-^13^C methyl correlation analysis.

### Correlation of analytical methods to the binding assays

With the exception of SEC, the analytical methods used to assess NISTmAb oxidation, *R*_2_(^1^H_2_O), 2D ^1^H-^13^C methyl gHSQC, Tycho, and SPR, all detected measurable changes within the first 4–5 h of chemical oxidation of Met residues, suggesting the possibility that the output from these analytical methods could be directly correlated. For the *R*_2_(^1^H_2_O) measurements, the exponential decay of Met resonance with increasing oxidation time is consistent with the reduction of Met signals observed in the high-field NMR spectra, although the *R*_2_(^1^H_2_O) plot levels off after 5 h, indicating the relaxation parameter sensitivity limit is reached by approximately 5 h of oxidation. As *R*_2_(^1^H_2_O) is readout from the bulk water, HOS perturbations need to influence the bulk solvent in order to induce an *R*_2_(^1^H_2_O) change. While the other analytical methods continue to detect HOS and stability changes beyond 5 h, visual correlation of *R*_2_(^1^H_2_O) with binding affinity from SPR measurements from early time points highly suggests that the functional integrity of NISTmAb is correlated to *R*_2_(^1^H_2_O). The *R*_2_(^1^H_2_O) measurement can therefore not only provide a real-time readout of change in HOS but can also be qualitatively correlated with overall drug substance (or, active pharmaceutical ingredient, API) integrity.

**Figure 8.**
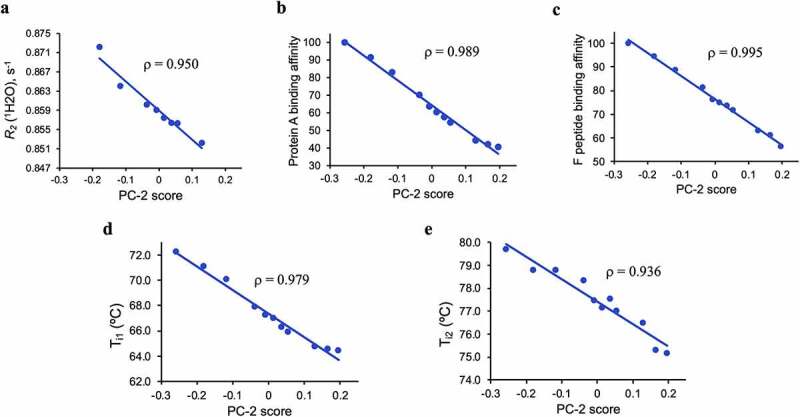
Linear correlation of PCA score to *R*_2_(^1^H_2_O), ligand binding and thermal stability analyses. Correlation plot of PC-2 score to: (a) *R*_2_(^1^H_2_O); (b) Protein A binding affinity; (c) F peptide binding affinity; (d) inflection temperature Ti_1_ of C_H_2 domain of Fc; (e) inflection temperature Ti_2_ of C_H_3 domain of Fc, where ρ corresponds to correlation constant. Note, correlation between *R*_2_(^1^H_2_O) and PC-2 in panel a is analyzed between 0.5 and 8 h.

To establish more quantitative correlations between 2D ^1^H-^13^C methyl gHSQC spectra, *R*_2_(^1^H_2_O) from *w*NMR, Tycho, and SPR results, correlation analyses were performed (Figure 8). The PC-2 scores from 2D NMR exhibit the greatest correlation with Protein A and F peptide binding changes (correlation coefficient, ρ, of 0.989 and 0.995), highlighting the ability of the PC-2 score to detect functionally relevant spectral changes from 2D ^1^H-^13^C methyl fingerprint spectra analysis (Figure 8b,c). Slightly reduced correlation was observed with the Tycho qualitative stability measurements versus the PC-2 score, from which a ρ of 0.979 and 0.936 for T_i1_ and T_i2_, respectively, were derived (Figure 8d,e). As the Tycho measurement is dependent on the solvent accessible state of tryptophan residues, the reduced ρ value for T_i2_ vs PC-2 score likely reflects that tryptophan solvent accessibility deviated slightly from a linear relationship with HOS changes. Although not for the entire duration of oxidation experiment, PC-2 scores also linearly correlate to *R*_2_(^1^H_2_O) measured in the early period (0.5–8 h) of the oxidation time course. As stated above, *R*_2_(^1^H_2_O), which is a measure of global exchange of protons between the mAb and bulk solvent, appears to be sensitive to the HOS perturbations observed early on in the time course of the oxidation, while the 2D NMR and other analytical and thermal stability methods and binding assays methods used sense both global and local changes. This suggests that HOS changes resulting from mAb oxidation follow a biphasic response with an initial global perturbation to HOS, which does not compromise the integrity of the mAb fold but is sensitive to the *w*NMR measurement, and localized HOS perturbations.

## Discussion

Analytical characterization of mAb-based therapeutics throughout clinical development is essential to ensure CQAs remain within established specifications.^42^ For the CQA of HOS, many low-to-moderate resolution biophysical methods are used without traceability to a high-resolution method. Further, functional assays (e.g., ELISA, SPR, bioassay) are used as an indirect HOS measurement of the structural integrity of a given product. Traceability to high-resolution methods such as NMR will enable more effective decision-making and may reduce the need for some lower resolution techniques.^43^ Further, high-field NMR methods are known to monitor multi-attributes simultaneously, including the ability to monitor subtle differences in HOS that are often missed by lower resolution methods.^43^ Herein, our study sought to link perturbations in 2D NMR spectra to a functional outcome and to correlate the high-resolution data to results from low-resolution analytical methods.

In particular, low-resolution *w*NMR served as an initial “on-line” assessment for real-time reaction monitoring. The signal source for this technology is the solvent, water. As hydrogens from water are in exchange with amides, amines, and hydroxyl groups from both the drug substance and excipients, the *w*NMR *R*_2_(^1^H_2_O) measurement provides an overall assessment of the physiochemical properties of the drug product. This technology has also proven effective for noninvasive measurements of biologics. The *R*_2_(^1^H_2_O) values have been successfully measured on unopened vials, which could then be used for clinical purposes.^44^ *w*NMR has been successfully applied to quality control on oils and oxidation-induced perturbations in the formation of fibrin clots; however, to our knowledge, this technology has not been applied to monitoring forced degradation mAb-based therapeutics. In this context, it has great potential due to simple and rapid nature of the measurement, with one *R*_2_(^1^H_2_O) measurement taking only approximately 50 seconds.

While *w*NMR gives an overall assessment of the drug product, it cannot provide the precise information provided by the 2D NMR method. The high-field NMR method underscores the ability to interrogate the HOS integrity of the mAb at atomic-level resolution. Even in the absence of residue-specific assignments, many resolved resonances can be traced to specific domains. Further, the PCA of the ^1^H-^13^C methyl correlation spectra without the Met and MetO regions reveal the extent to which the HOS around each oxidized Met residue became perturbed. The correlation of the 2D NMR PCA with the SPR binding affinity underscores that an observed spectral change can be associated with a critical degradation of protein function. As such, with the traceability to a functional outcome, we anticipate that spectral changes in the 2D NMR can inform decisions about the continued developability of a drug product.

The implementation of 2D NMR with other methods in forced degradation studies of mAb candidates will help address US Food and Drug Administration guidelines to use state-of-the-art analytical techniques and orthogonal quantitative methods to definitively identify differences in therapeutic product attributes.^5^ The capability of the NMR fingerprinting method to simultaneously detect and quantify both the unmodified and oxidized Mets further demonstrates the advantage of using 2D NMR for accurate detection of degradation products that result from post-translational modifications per regulatory recommendations.^4,10^ Furthermore, the sensitivity to low levels of mAb modifications makes 2D NMR a suitable technique for comparability studies of biosimilar candidates and originator or reference drugs. While the ^1^H,^13^C gHSQC spectrum took 3.7 h of experimental time in this study, newer pulse programs have become available to reduce the measurement time down to 1 h using the extra alternative large band selective optimized-flip-angle short-transient pulse sequence.^45^ In addition to precise detection of modified residues and other spectral perturbations, the direct correlation of 2D NMR data to other analytical methods fulfills the validation criterion of a robust methodology for CQA assessment of biosimilars.^46^

Taken together, the *w*NMR and 2D NMR methods as demonstrated through this study provide robust and versatile tools to monitor mAb oxidation from the global to local HOS perspective, allowing the real-time monitoring of *R_2_*(^1^H_2_O) to be correlated with detailed assigned atomic-level structural responses that can be correlated with mAb stability and binding functionality.

## Materials and methods

### Oxidation protocol

Samples were prepared from NISTmAb Primary Sample (PS)-8670 stock of 100 mg/mL in 25 mM L-histidine pH 6.0 and 30% hydrogen peroxide (Sigma-Aldrich). NISTmAb (40 mg/mL, 270 µM) in 25 mM L-histidine pH 6.0 was oxidized with 0.3% (v/v) hydrogen peroxide in 8.1 mL total volume using an 18-mm borosilicate glass tube (Fisher Scientific). Note that an optimized real-time, *w*NMR measurement protocol is outlined below; however, intensive optimization was required to obtain repeatable results. The Supplementary Information includes details of this optimization process (**Supplementary Methods, Figures S1–S6**).

The benchtop NMR was equilibrated to 25°C. Hydrogen peroxide was added last, and the sample was mixed by pipetting with a serological pipet 5 times. The reaction mixture was incubated in a time-domain benchtop NMR instrument (MQC+, Oxford Instruments) at 25°C, and water T_2_ was recorded every 15 min for 24 h with the tube left open. Aliquots of 0.77 mL were removed after 0.5, 1, 2, 2.5, 3, 3.5, 4, 8, 12, and 24 h of oxidation and immediately transferred to a 2-mL tube containing buffer equilibrated 30-µL catalase cross-linked agarose beads (Sigma Aldrich) to quench the oxidation reaction. Each aliquot was quenched for 20 min, at room temperature, while mixing and gently vortexing tube every 3 min. After decanting, the quenched sample was separated into 5 portions, 1 × 600 μL and 4 × 25 μL. The 25-μL aliquots were diluted to 10 mg/mL with 25 mM L-histidine, and all aliquots were stored at −80°C until further analysis. A reference unoxidized NISTmAb sample was also treated with the oxidation quenching procedure to serve as a control.

Water T_2_ was also recorded for the oxidation of NISTmAb supplemented with 1 mM ethylenediaminetetraacetic acid (EDTA) in a total volume of 3.7 mL without aliquot removal. Samples were mixed via Y-shaped apparatus, which was prepared in-house by connecting an 8-inch fluorinated ethylene propylene tubing (i.d.: 1/16^”^) with two 3-mL syringes via a three-way connector (McMaster-Carr) (**Figure S2**). Equivalent volume, 1.85 mL, of diluted hydrogen peroxide solution (0.6% (v/v) in 25 mM L-histidine, 1 mM EDTA) and NISTmAb (80 mg/mL in 25 mM L-histidine, 1 mM EDTA) were loaded into the two separate 3-mL syringes and simultaneously added into 18 mm NMR tube using the Y-shaped tubing to ensure mixing of NISTmAb with hydrogen peroxide. The tube was immediately inserted to the NMR instrument probe, and T_2_ was recorded every 15 min for 24 h. The average water T_2_ values were determined from two replicate oxidation experiments.

### Time-domain *w*NMR and processing

Oxidation kinetics was monitored via the changes in water proton transverse relaxation rate *R*_2_(^1^H_2_O) over 24 h with data collection every 15 min (91 data points total). Water proton transverse relaxation data were collected using time-domain benchtop NMR instrument MQC+ (Oxford Instruments plc, UK),^1^H resonance frequency 23.8 MHz (0.56 T), probe ID 26 mm, magnet and probe temperature 25°C. Echo signal decay was obtained using conventional Carr–Purcell–Meiboom–Gill (CPMG) pulse sequence 90°(*x*)-*τ*-180°(*y*)-*τ.^47^*

Total number of accumulations was 2, at interpulse delay (*τ*) 500 μs. Relaxation delay between accumulations was set to 12 s, and the numbers of echoes collected was 12,000. The duration of a single measurement was ~50 s.

Resulting echo signal decay data were brought in phase using RINMR ver. 7.0 software (Oxford Instruments plc, UK) and exported to three-column format (time, real and imaginary signal intensity) for further processing to extract water proton transverse relaxation rate *R*_2_(^1^H_2_O) values. *R*_2_(^1^H_2_O) was calculated using automated batch processing with NMRPipe^48^ scripts.

### 2D NMR experiments and processing

NMR samples were prepared from the 600 μL oxidation quenched aliquot that were thawed on the day of data collection and supplemented with 3% D_2_O and 150 M deuterated 3-(trimethylsilyl) propane-1-sulfonate (DSS-d_6_) (Sigma Aldrich). Samples were loaded into 5 mm standard NMR tubes for data acquisition. NMR experiments were measured at 50°C on a Bruker Avance III 600 MHz spectrometer equipped with a triple resonance cryogenetically cooled TCI probe and *z*-axis axis gradient system. 2D NMR data were recorded using a gradient-selected, sensitivity enhanced ^1^H- ^13^C gHSQC experiment with a recycle delay of 1.5 s.^49^ Data were collected with 64 scans and 128 increments and acquisition times of 100 ms and 14 ms in the direct and indirect dimension, respectively. Spectral window of 14 and 30 ppm, corresponding to a total data matrix of 1682 × 128 total points. Five replicate experiments were collected for each oxidation time point aliquot over the course of 18.5 h. Each experiment took approximately 3.7 h.

Spectra were processed using NMRPipe.^48^ An automated batch processing scheme was used to execute NMRPipe scripts as previously described.^50^ Spectra were aligned and normalized according to the maximum value of the DSS reference signal. The processed data were interpolated to generate spectra that have identical size and chemical shift range. PCA was performed without centering. The methyl region ranged from ^1^H 2.25 to −0.65 ppm and ^13^C 28.0 to 18.9 ppm using pcaNMR function of NMRPipe.^48^ pcaNMR uses NIPALS algorithm to calculate principal components and generate corresponding spectra for each component, known as loading plots.^48,51^ The resulting PCA scores were imported to MATLAB to perform cluster analysis and to generate score plots. Peak integrals of Met and MetO regions were generated by first summing over replicate measurements and then dividing by the summation of the DSS reference region for that spectrum.

### Thermal unfolding

The thermal stability of oxidized NISTmAb samples was evaluated using intrinsic fluorescence measurement with a Tycho NT.6 instrument (Nanotemper Technologies). The instrument measured intrinsic fluorescence of tryptophan at both 350 and 330 nm as the 10 mg/mL oxidation series samples were heated from 35°C to 95°C in 10 μL capillaries at a rate of 30°C/min. The NanoTemper software generated an unfolding profile by plotting the 350/330 nm ratio as a function of temperature. The first derivative of this plot was then taken, and the inflection temperatures, T_i_, determined from the maximum and minimum peaks. The average value of inflection temperatures was calculated from two replicate experiments.

### SEC analysis

SEC was performed as previously described.^35^ Samples were analyzed on Thermo Scientific Dionex U3000 high-pressure LC system using Acquity UPLC Protein BEH SEC column (Waters Corporation, 1.7 µm particle size, 200 Å pore size, 4.6 × 150 mm length) and 100 mM Sodium phosphate, 250 mM sodium chloride pH 6.8 solution. Then, 6 μL sample of 10 mg/mL was injected onto the preconditioned column at flow rate of 0.3 mL/min and peak detection was monitored at 280 nm. Chromatograms were processed using the Dionex Chromeleon 7 software (Thermo Scientific).

### Ligand binding analysis

SPR analysis was carried out on a Biacore T200 system (GE Healthcare) using Series S Sensor Chip CAP to immobilize the ligands. Series S Sensor Chip CAP, PBS-P+ buffer and Biotin capture Kit were all purchased from GE Healthcare. Recombinant biotinylated Protein A (29989) and Protein L (21189) were purchased from Thermo Fisher. A peptide epitope of the NISTmAb, F peptide, with the sequence NSELLSLINDMPITNDQKKLMSNN and N-terminal acetylation, C-terminal amidation, and a C-terminal biotinylated lysine residue was previously synthesized by Genscipt.^18^

Binding measurements were performed following a previously developed method.^18^ Briefly, biotinylated ligands (Protein A, Protein L, F peptide) were immobilized to a CAP sensor chip via biotin CAPture reagent consisting of streptavidin bound to an oligonucleotide that is complementary to the strand on the sensor chip. Protein A and Protein L ligands were diluted to 0.02 μg/mL with the running buffer, PBS pH 7.4, and supplemented with 0.1% BSA while F peptide was diluted to 0.05 μg/mL. Biotin capture reagent was injected at a flow rate of 2 μL/min for 300 s followed by the injection of biotinylated ligands via three separate flow channels, Fc2 (Protein A), Fc3 (F peptide), Fc4 (Protein L), at 5 μL/min flow rate for 60 s to immobilize Protein A, F peptide and Protein L. Samples, which were diluted to 200 nM with the running buffer, were loaded to each flow channel at a flow rate of 50 μL/min and allowed to associate for 100 s and dissociate for 300 s. The maximum binding level at the end of the association phase was used as the binding affinity response value for each sample. The binding affinity response values were normalized by dividing with the maximum value of the NISTmAb reference for each ligand. The relative binding affinity of each sample was calculated from the average of two independent experiments.

## Supplementary Material

Supplemental Material

## Abbreviations

CQA critical quality attribute

DSS-d_6_ deuterated 3-(Trimethylsilyl) propane-1-sulfonate

gHSQC gradient-selected, sensitivity enhanced heteronuclear single-quantum coherence

HOS higher order structure

Ile isoleucine

LC liquid chromatography

mAb monoclonal antibody

Met methionine

MetO methionine sulfoxide

NMR nuclear magnetic resonance spectroscopy

PCA principal component analysis

ROS reactive oxygen species

SEC size-exclusion chromatography

SPR surface plasmon resonance

*R*_2_(^1^H_2_O) water proton transverse relaxation rate

*w*NMR water nuclear magnetic resonance spectroscopy
