# Supplementary material for: Correlated analytical and functional evaluation of higher order structure perturbations from oxidation of NISTmAb
Source: MAbs. 2023 Jan 22;15(1):2160227. doi: 10.1080/19420862.2022.2160227 (PMC9872951; doi:10.1080/19420862.2022.2160227)
Supplement: Supplemental Material [file KMAB_A_2160227_SM1988.docx]

**Supplementary Information for:**

**Correlated Analytical and Functional Evaluation of Higher Order Structure Perturbations from Oxidation of NISTmAb**

Tsega L. Solomon^1^, Frank Delaglio^1^, John P. Giddens^1^, John P. Marino^1^, Yihua Bruce Yu^2^, Marc B. Taraban^2^, Robert G. Brinson^1*^

^1^Institute for Bioscience and Biotechnology Research, National Institute of Standards and Technology and the University of Maryland, Rockville, MD 20850

^2^Bio- and Nano-Technology Center, University of Maryland School of Pharmacy, and Institute for Bioscience and Biotechnology Research, Rockville, MD 20850

*Keywords: low field water NMR relaxometry, 2D NMR, biologics, NISTmAb, higher order structure, surface plasmon resonance, antigen binding*

*Corresponding Author

Robert G Brinson, Ph.D.

Institute for Bioscience and Biotechnology Research

National Institute of Standards and Technology

9600 Gudelsky Drive

Rockville, Maryland 20850

United States

E-mail: [robert.brinson@nist.gov](mailto:robert.brinson@nist.gov)

**Supplemental Methods**

***Development of the protocol to monitor NISTmAb oxidation via water proton NMR transverse relaxation***

In this study we demonstrated the utility of water-proton transverse relaxation rate, *R_2_*(^1^H_2_O), for monitoring the oxidation in accelerated stability study of therapeutic mAb. However, the optimized experimental protocol was realized only after extensive method development which revealed the sensitivity of *w*NMR technique to variances, often subtle, in sample preparation. Here, we describe experimental considerations that need to be taken into account when monitoring an oxidation reaction by *R_2_*(^1^H_2_O) in a low-field benchtop instrument. The troubleshooting process for a reproducible procedure led to the discovery of several factors that can impact *R_2_*(^1^H_2_O), including sample mixing, aliquot removal, and the presence of trace metals.

During the initial attempts at monitoring the NISTmAb oxidation reaction, the sample was mixed by pipetting after the addition of H_2_O_2_, and *R_2_*(^1^H_2_O) was recorded every 15 minutes while aliquots were removed at different time points during the 24 hours oxidation period. The resulting *R_2_*(^1^H_2_O) plot (**Figure S1A**) showed variability throughout the 24 hours where *R_2_*(^1^H_2_O) decreased from 0.93 s^-1^ to 0.87 s^-1^ in the first 1.5 hours, stabilized at 0.87 s^-1^ between 1.5 hours and 8 hours, followed by a staggered increase to nearly 0.92 s^-1^ from 8 hours to 24 hours. This change in *R_2_*(^1^H_2_O) however did not correlate with the time dependent changes measured by 2D NMR, thermal stability or ligand binding assay of the aliquoted samples from the same reaction mixture. The inconsistency of *R_2_*(^1^H_2_O) measurements with the results from the orthogonal methods suggested that the *w*NMR measurement was influenced by experimental factors other than perturbations to mAb HOS. In the *w*NMR method, *R_2_*(^1^H_2_O) was recorded in the presence of H_2_O_2_ for the duration of the oxidation reaction while the other orthogonal measurements were recorded after quenching the oxidation reaction and removing excess H_2_O_2_. Therefore, external factors from sample handling can influence *R_2_*(^1^H_2_O) measurement during reaction monitoring and result in the *w*NMR method detecting experimental variance in addition to change in mAb HOS. A similar inconsistent change in *R_2_*(^1^H_2_O) plot was also observed for the control oxidation of L-histidine buffer in the absence of mAb, further indicating the need to optimize the sample preparation procedure (**Figure S1B**).

***Optimized sample preparation***

To minimize the introduction of external oxygen during NISTmAb and H_2_O_2_ mixing by pipetting, the influence of volume change from aliquot removal, and to ensure the uniform mixing of the reactants, a Y-shaped apparatus was employed for sample mixing (**Figure S2**) where NISTmAb and H_2_O_2_ solutions were simultaneously loaded into the benchtop NMR tube, and *R_2_*(^1^H_2_O) was recorded without aliquot removal. While the *R_2_*(^1^H_2_O) plot was less noisy than the initial oxidation reaction, an inconsistent change in *R_2_*(^1^H_2_O) was still detected (**Figure S3A**) with a decrease from 0.90 s^-1^ to approximately 0.86 s^-1^ in the first 2 hours of oxidation followed by a gradual increase to 0.92 s^-1^ from 2 hours to 24 hours. This variance in *R_2_*(^1^H_2_O) again did not correlate with the time dependent change of the other analytical methods. Three replicate control oxidation experiments with L-histidine buffer using the same mixing technique and without aliquot removal further yielded irreproducible *R_2_*(^1^H_2_O) plots (**Figure S3B**) with varying magnitude of change in *R_2_*(^1^H_2_O).

The inconsistent drift in *R_2_*(^1^H_2_O) prompted an additional control study to establish the reproducibility of the benchtop *w*NMR instrument by measuring *R_2_*(^1^H_2_O) of the L-histidine buffer in the absence of H_2_O_2_. The resulting replicate *R_2_*(^1^H_2_O) plot (**Figure S4**) showed slight change in *R_2_*(^1^H_2_O) in the first 30 minutes, which was attributed to temperature equilibration, and a steady *R_2_*(^1^H_2_O) for the 24 hours of data collection. This result signified the reproducibility of the *w*NMR instrument and suggested that the variation observed in the control oxidation experiments is due to the oxidizing agent, H_2_O_2_.

**Reproducible oxidation with addition of EDTA**

It has been documented that transition metals, such as iron or copper, can react with hydrogen peroxide to produce hydroxyl radicals and/or other ROS.^1^ In the present study, trace metals such as iron could be introduced into the reaction mixture from air, buffer or the reaction container. This led us to hypothesize that trace metals may be contributing to the decomposition of H_2_O_2_ that could in turn affect the relaxation property of water protons and lead to the observed *R_2_*(^1^H_2_O) variability, since we cannot control the concentration of these metal ions coming from different sources. To test this hypothesis, 1 mM EDTA was added during replicate L-histidine buffer control oxidation experiments, while using the Y-shaped apparatus for mixing. The *R_2_*(^1^H_2_O) plots of buffer oxidation replicates were relatively constant after the initial equilibration period of 30-minutes for two replicate experiments (**Figure S5A**) and similar to the condition in the absence of H_2_O_2_ (**Figure S4**). This optimized condition was implemented for NISTmAb oxidation, where 1 mM EDTA was added to the solution of NISTmAb and H_2_O_2_ prior to sample mixing via Y-shaped apparatus (**Figure S5B**). The *R_2_*(^1^H_2_O) plot of replicate NISTmAb oxidation experiments showed exponentially decaying *R_2_*(^1^H_2_O) change within the first 5 hours of oxidation and variability that was in the noise after 5 hours. The result demonstrated that reproducible *R_2_*(^1^H_2_O) measurements related to the property of the mAb could be made by supplementing the oxidation reaction with EDTA.

To examine whether the optimized procedure and the addition of EDTA affects the efficiency of H_2_O_2_ to oxidize NISTmAb, 2-hour and 24-hour aliquots from the optimized sample preparation were assessed by 2D NMR. The spectral overlay of 2-hour and 24-hour aliquots in the presence and absence of EDTA and closer examination of the Met and MetO regions in the two-hour aliquots afforded closely matching spectral alignment (**Figure S6**), indicating that EDTA had minimal effect on the extent of the H_2_O_2_ oxidizing activity on NISTmAb. Thus, the addition of EDTA in the oxidation reaction mixture eliminated variability in *R_2_*(^1^H_2_O) by inhibiting the inconsistent interaction of H_2_O_2_ with contaminating trace metals. In addition to monitoring an oxidation reaction, the unanticipated variance in *R_2_*(^1^H_2_O) to the reaction conditions reflects the high sensitivity of *w*NMR to sample variability. These results further suggest that TD-NMR may be used for other reaction monitoring purposes of other forced degradation methods. Further studies are needed, but TD-NMR may have the potential for general use in pharmaceutical industry to monitor operational inconsistency and therapeutic variability.

**Supplementary Figures**

**
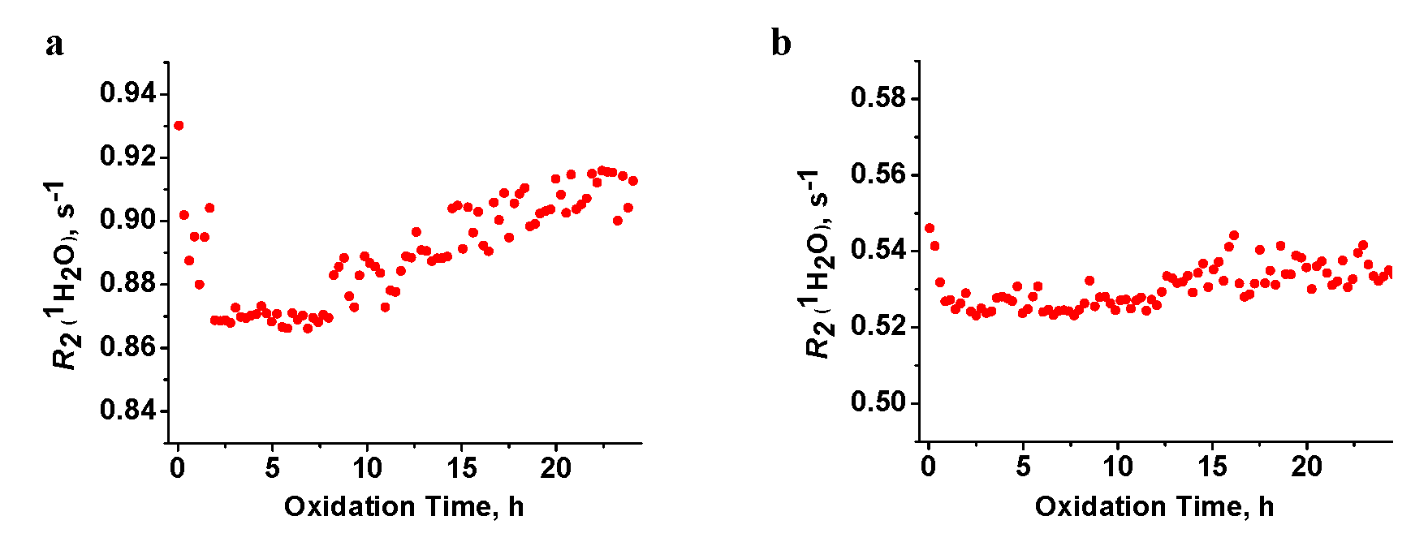
**

**Figure S1.** Initial results prior to process optimization: Monitoring *R_2_*(^1^H_2_O) as aliquots are removed during oxidation of NISTmAb and buffer. (a) *R_2_*(^1^H_2_O) during the course of NISTmAb oxidation with 0.3% H_2_O_2_ in 25 mM L-histidine pH 6.0 buffer. (b) *R_2_*(^1^H_2_O) during the course of only 25 mM L-histidine pH 6.0 buffer oxidation with 0.3% H_2_O_2_. All aliquots were removed at the following time points: 0.5, 1, 2, 2.5, 3, 3.5, 4, 8, 12, 24 hours.

**Figure S1 Alt Text**. Initial experiments measuring the water traverse relaxation rate by time domain nuclear magnetic resonance spectroscopy showed noisy data and poor baselines.


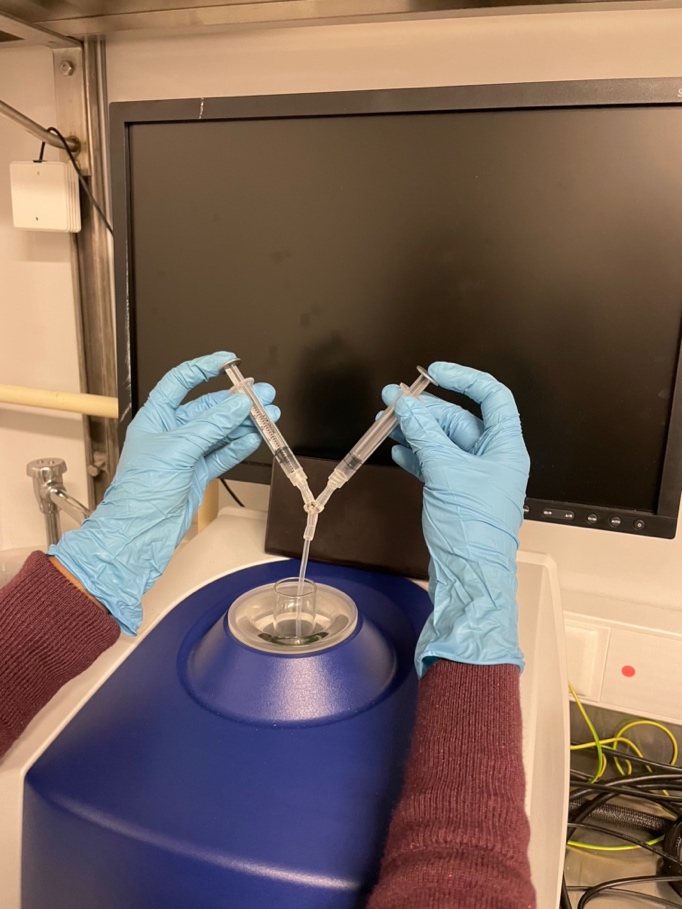


**Figure S2.** Photo of Y-shaped apparatus used for mixing NISTmAb with hydrogen peroxide during sample loading into 18 mm tube for water *T_2_* measurement in NMR benchtop NMR instrument.

**Figure S2 Alt Text**. Sample mixing and loading into the benchtop NMR instrument involved a Y-shaped apparatus.


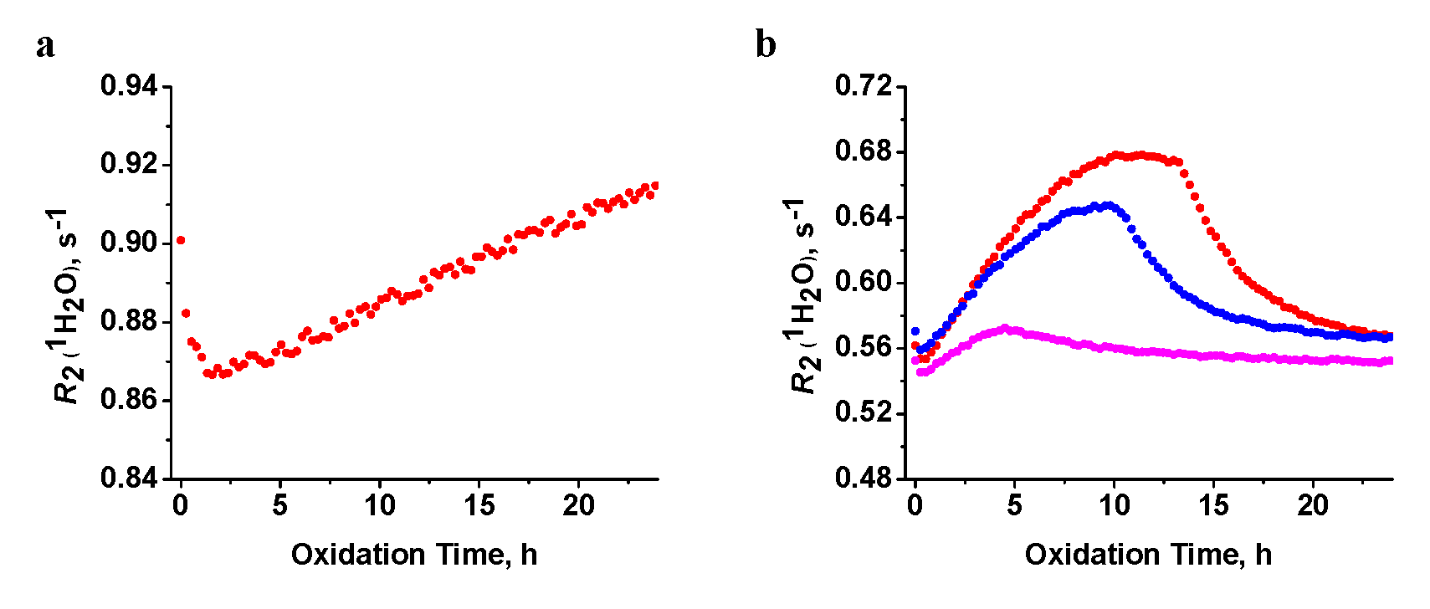


**Figure S3.** Water transverse relaxation rate, *R_2_*(^1^H_2_O), of NISTmAb and buffer without aliquot removal. (a) *R_2_*(^1^H_2_O) during the course of NISTmAb oxidation with 0.3% H_2_O_2_ in 25 mM L-histidine pH 6.0 buffer. (b) Three replicate measurements of *R_2_*(^1^H_2_O) during control oxidation study with 25 mM L-histidine pH 6.0 buffer oxidation.

**Figure S3 Alt Text**. Additional water transverse relaxation controls with hydrogen peroxide and buffer only or NISTmAb only, both without aliquot removal, afford irreproducible data.

**Figure S4.** Monitoring the repeatability of *R_2_*(^1^H_2_O) measurement. Replicate measurement of *R_2_*(^1^H_2_O) of 25 mM L-histidine buffer (pH 6.0) in the absence of H_2_O_2_.

**Figure S4 Alt Text**. Control water transverse relaxation rate measurements with only buffer in the absence of hydrogen peroxide afford repeatable baselines.


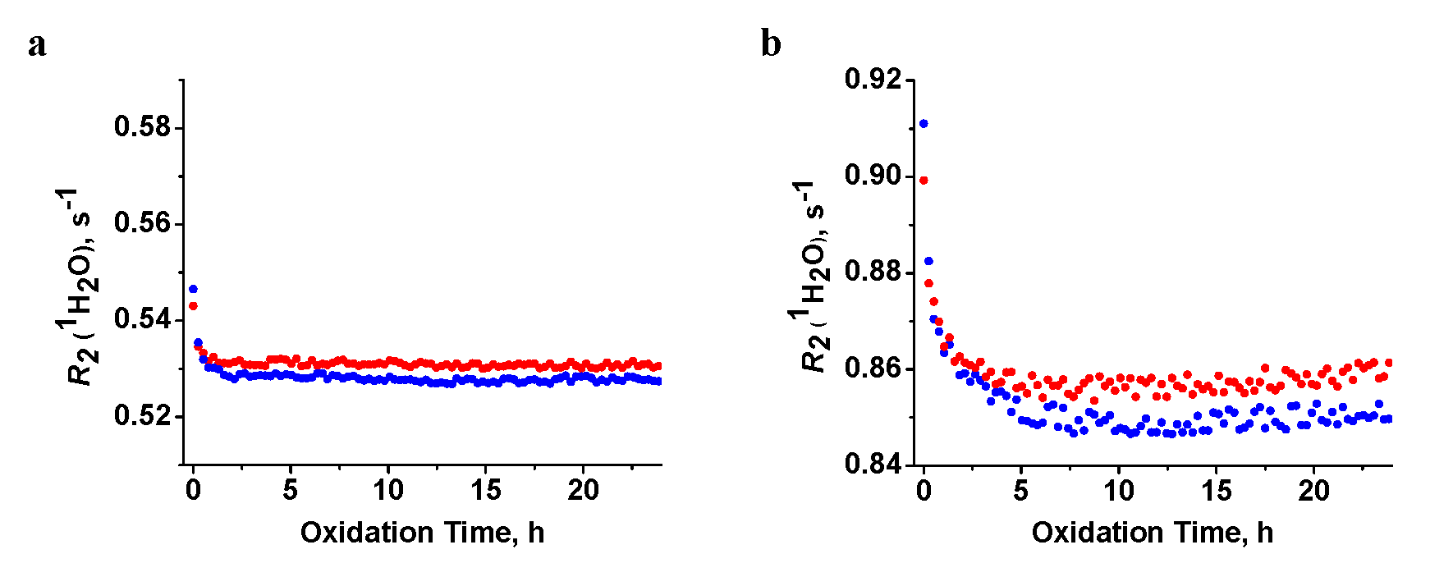


**Figure S5.** Repeatable *R_2_*(^1^H_2_O) measurement during buffer and NISTmAb oxidation without aliquot removal. (a) Plot of replicate *R_2_*(^1^H_2_O) measurement of 25 mM L-histidine buffer (pH 6.0) in the presence of 0.3% H_2_O_2_ and 1 mM EDTA. (b) Plot of replicate *R_2_*(^1^H_2_O) measurement during the incubation of NISTmAb with 0.3% H_2_O_2_ in the presence of 1 mM EDTA.

**Figure S5 Alt Text**. Addition of EDTA acid to the oxidation reaction affords repeatable results for the water transverse relaxation measurement.


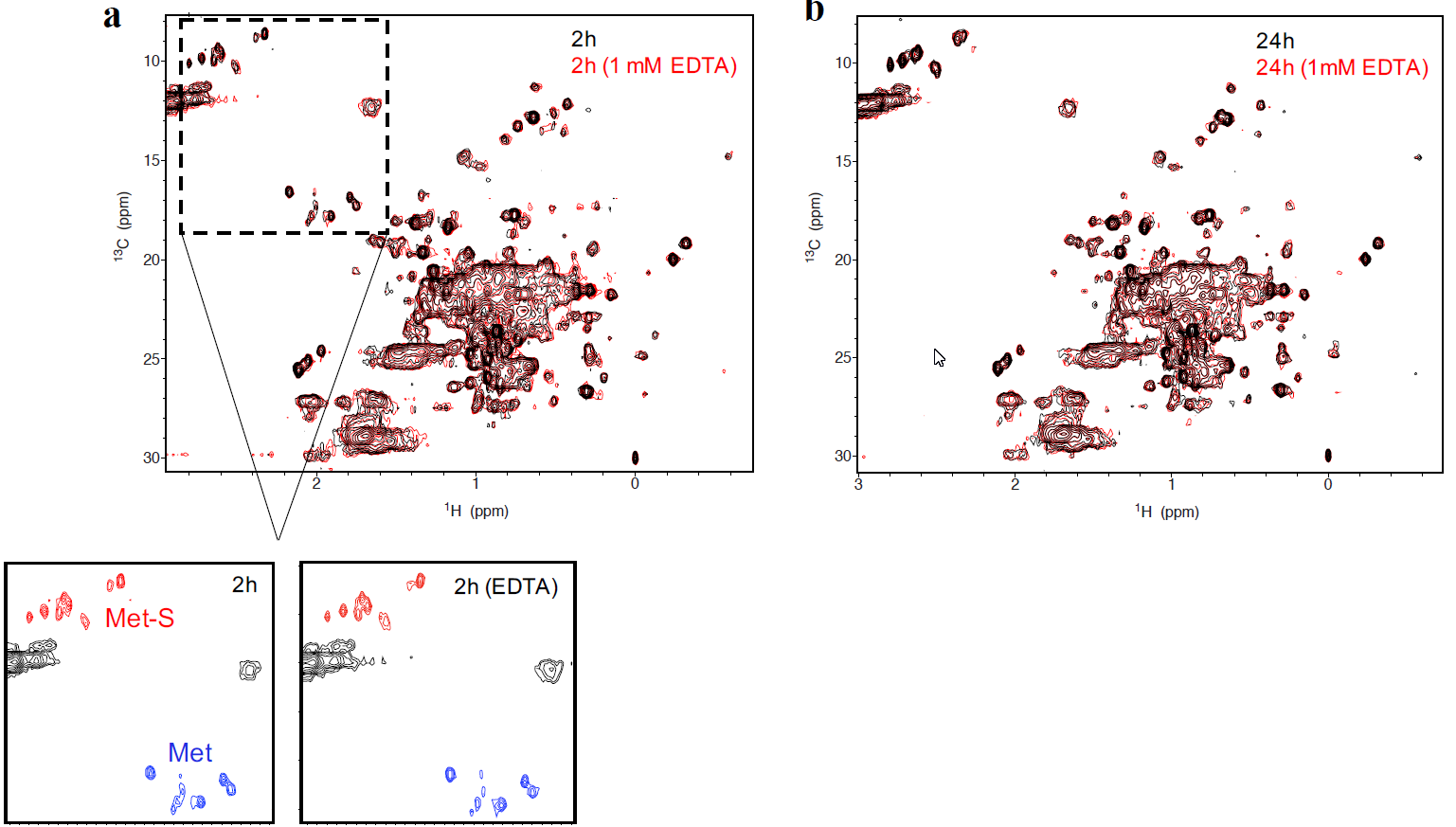


**Figure S6.** Comparison of NISTmAb oxidation in the presence and absence of EDTA via 2D ^1^H-^13^C Methyl HSQC spectra. (a). Overlay of the 2D ^1^H-^13^C Methyl HSQC spectra of 2 hours oxidized NISTmAb without EDTA (black) with 1 mM EDTA (red). The methionine and methionine sulfoxide regions of the ^1^H-^13^C Methyl HSQC spectra of the 2 hours oxidized NISTmAb are shown below the full Methyl spectra. (b) Overlay of the 2D ^1^H-^13^C Methyl HSQC spectra of 24 hours oxidized NISTmAb without EDTA (black) with 1 mM EDTA (red).

**Figure S6 Alt Text**. High field 2D NMR fingerprinting shows similar spectral output with and without the addition of EDTA acid during the oxidation reaction.


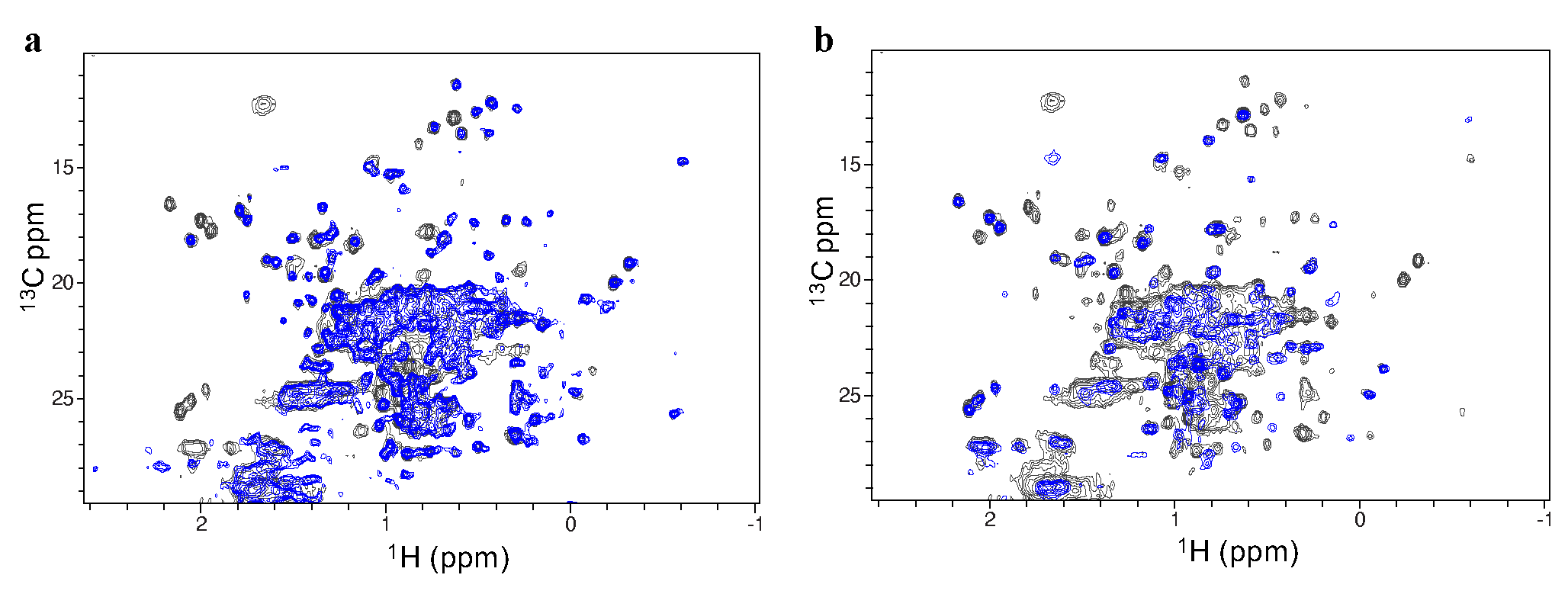


**Figure S7.** Alignment of intact NISTmAb 2D methyl fingerprint spectra to its Fab and Fc fragments. (a) Overlay of the 2D ^1^H-^13^C Methyl gHSQC spectra of NISTmAb reference (gray) and NISTmAb-Fab fragment (blue). (b) Overlay of the 2D ^1^H-^13^C Methyl gHSQC spectra of NISTmAb reference (gray) and NISTmAb-Fc fragment (blue). Spectra collected at 600 MHz and 50°C.

**Figure S7 Alt Text**. Alignment of the high field 2D methyl fingerprints from the oxidation time course to the respective methyl fingerprints of each Fab and Fc domain allow identification of domain-specific signals.


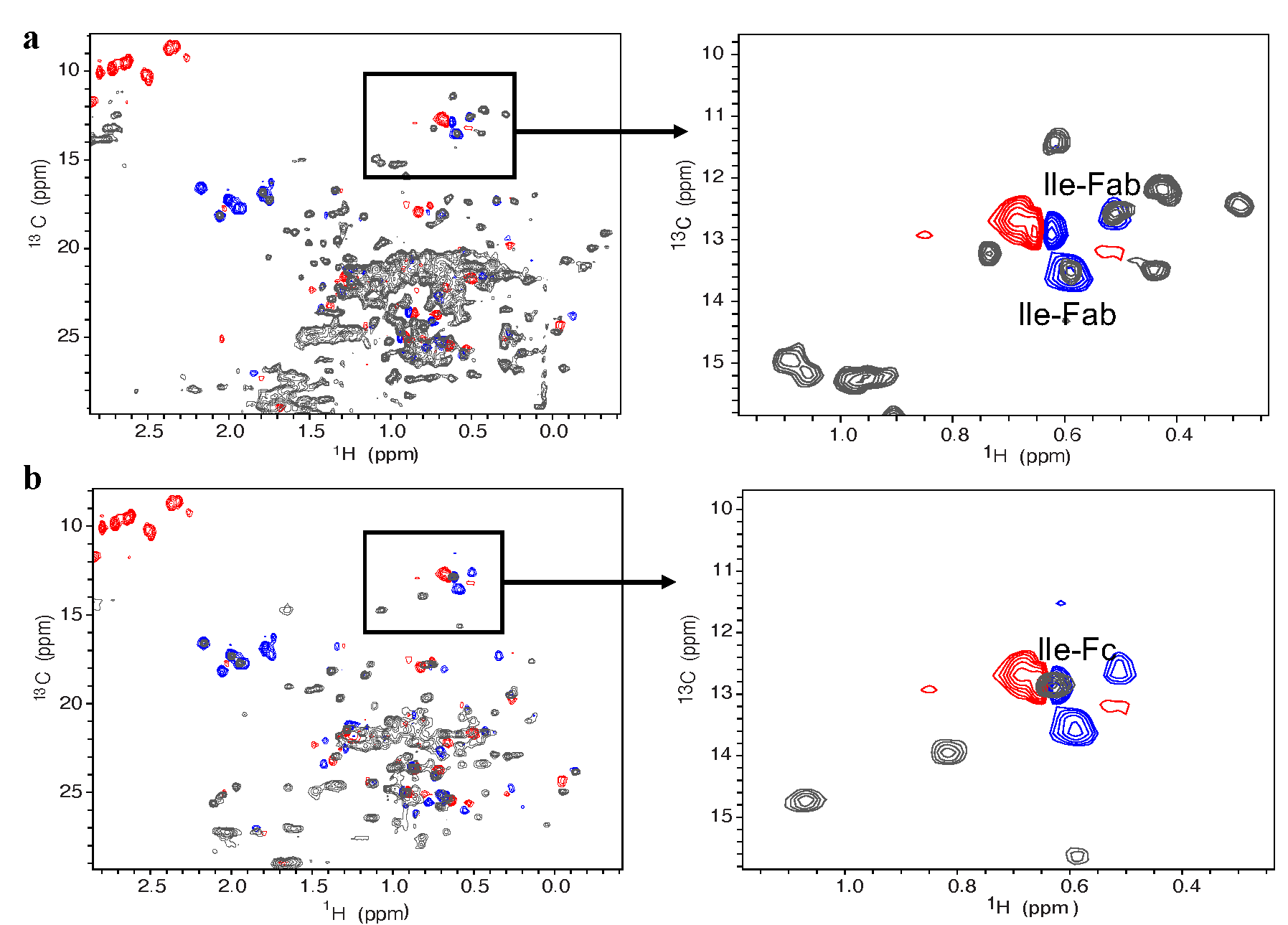


**Figure S8.** Variance in PC-2 loading spectra corresponds to signals in both the Fab and Fc fragments. (a) The spectral loading plot of the second component, PC-2, (red and blue contours) overlaid on the 2D ^1^H-^13^C methyl gHSQC spectrum (gray) of the Fab fragment with Ile region boxed (left) and expanded view (right). (b) The spectral loading plot of the second component, PC-2, (red and blue) overlaid on the 2D ^1^H-^13^C methyl gHSQC spectrum (gray) of the Fc fragment with Ile region boxed (left) and expanded view (right).

**Figure S8 Alt Text**. Overlay of the spectral loading plots with the 2D methyl fingerprints of each domain allows for domain specific assignment of isoleucines whose chemical shifts have been perturbed from the oxidation.


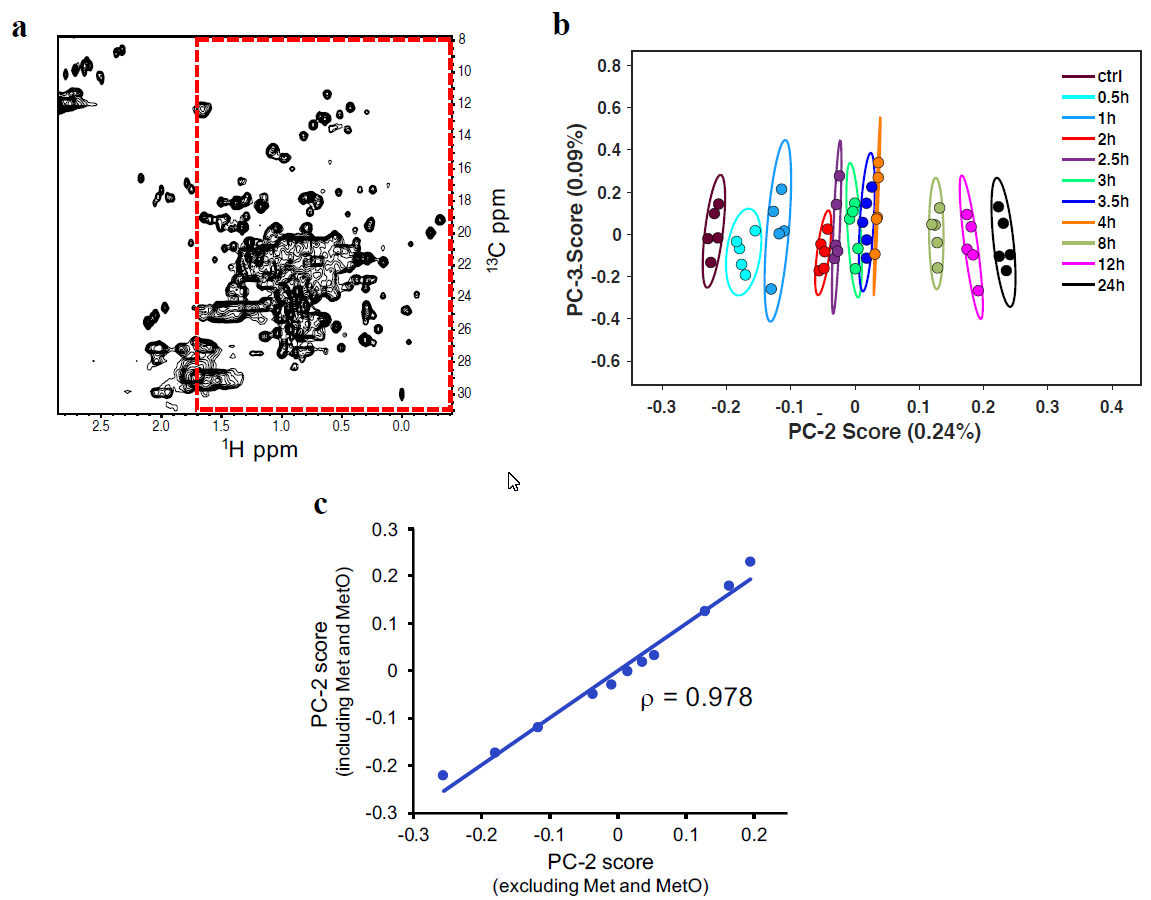


**Figure S9.** PCA of 2D ^1^H-^13^C methyl gHSQC spectra based on oxidation time course with the Met and MetO regions excluded. (a) The PC-1 loading plot of the methyl region from the 2D ^1^H-^13^C methyl gHSQC spectra of all the oxidation time point aliquots, which consist of 5 replicate experiments for each time point aliquot. The red boxed region excluding Met and MetO signals was used as input for PCA. (b) Score plot of PC-2 and PC-3 from analysis of the methyl region excluding Met and MetO region. Replicate experiments have the same color. The ellipses correspond to the 95% confidence interval of the spread for each cluster. (c) Correlation plot of PC-2 scores of the entire methyl region shown in Panel a versus the region boxed in red excluding Met and MetO signals.

**Figure S9 Alt Text**. The score plots from principal component analysis of the 2D methyl fingerprints are highly similar, whether the chosen spectral region includes or excludes the methionine and methionine sulfoxide signals.

**Figure S10.** The binding sites of Protein A (blue), Protein L (green) and F peptide (orange) are circled on the crystal structure of the Fab (PDB 5K8A) and Fc (PDB 5VGP) domains. Met residues (magenta) are shown in sphere representation.

**Figure S10 Alt Text**. The regions where Protein A and the F peptide bind to NISTmab are proximal to methionine residues. The region on NISTmAb where Protein L binds is distal from any methionine.

**Table S1.** SEC analysis of NISTmAb oxidation series at 25°C.

|  | Monomer (%) | HMW^a^ (%) | LMW^b^ (%) |
| --- | --- | --- | --- |
| Control | 98.8 | 1.0 | 0.2 |
| 0.5h | 98.9 | 0.9 | 0.2 |
| 1h | 98.9 | 0.9 | 0.2 |
| 2h | 99.0 | 0.8 | 0.2 |
| 2.5h | 99.0 | 0.8 | 0.2 |
| 3h | 99.0 | 0.8 | 0.2 |
| 3.5h | 99.0 | 0.8 | 0.2 |
| 4h | 99.0 | 0.8 | 0.2 |
| 8h | 99.1 | 0.7 | 0.2 |
| 12h | 99.1 | 0.7 | 0.2 |
| 24h | 99.1 | 0.7 | 0.2 |
| ^c^PS 8670^2^ | 98.78 ± 0.12 | 1.02 ± 0.12 | 0.20 ± 0.01 |

^a^High molecular weight species (dimer and trimer) relative abundance

^b^Low molecular weight fragments relative abundance

^c^Literature values for NISTmAb reference (PS-8670) with standard uncertainty.^2^ In reference 2, the values were reported to 2 decimal places.

**Table S1 Alt Text**. Size Exclusion Chromatography afforded no statistically significant higher or lower molecular weight species of NISTmAb from the oxidation time course.

**Supplementary References**

1. Goldstein S, Meyerstein D, Czapski G. The Fenton reagents. Free Radic Biol Med 1993; 15:435-45.

2. Turner A, Yandrofski K, Telikepalli S, King J, Heckert A, Filliben J, et al. Development of orthogonal NISTmAb size heterogeneity control methods. Anal Bioanal Chem 2018; 410:2095-110.
